# Pain Management Mobile Applications: A Systematic Review of Commercial and Research Efforts

**DOI:** 10.3390/s23156965

**Published:** 2023-08-05

**Authors:** Yiannis Koumpouros, Aggelos Georgoulas

**Affiliations:** 1 Department of Public and Community Health, School of Public Health, Athens Campus, University of West Attica, 11521 Athens, Greece; 2 Department of Informatics and Computer Engineering, School of Engineering, Egaleo Park Campus, University of West Attica, 12243 Egaleo, Greece

**Keywords:** mobile health (mhealth), pain, information and communication technologies, ehealth, pain management, pain assessment

## Abstract

Shared decision making is crucial in the pain domain. The subjective nature of pain demands solutions that can facilitate pain assessment and management. The aim of the current study is to review the current trends in both the commercial and the research domains in order to reveal the key issues and guidelines that could further help in the effective development of pain-focused apps. We searched for scientific publications and commercial apps in 22 databases and the two major app stores. Out of 3612 articles and 336 apps, 69 met the requirements for inclusion following the PRISMA guidelines. An analysis of their features (technological approach, design methodology, evaluation strategy, and others) identified critical points that have to be taken into consideration in future efforts. For example, commercial and research efforts target different types of pain, while no participatory design is followed in the majority of the cases examined. Moreover, the evaluation of the final apps remains a challenge that hinders their success. The examined domain is expected to experience a substantial increase. More research is needed towards the development of non-intrusive wearables and sensors for pain detection and assessment, along with artificial intelligence techniques and open data.

## 1. Introduction

Chronic pain is a widespread, complex, and distressing issue that has a profound effect on people and society [1]. According to the European Pain Federation, more than 500 million days of missed work in Europe were caused by chronic pain; this can be translated into more than EUR 300 billion (1.5–3% of the gross domestic product) [2]. In 2019, recognizing its importance, the World Health Organization’s International Classification of Diseases (ICD-11) incorporated a new classification system of chronic pain [3]. Each person may feel pain differently. Thus, a significant issue of subjectivity arises. At the same time, clinicians need objective data and methodologies to support their decisions and reach a proposed therapy. In order to address the many challenges associated with pain, novel approaches are needed (i.e., assessment and treatment).

A key element of patient-centered care is patient empowerment. In a new patient-centered care paradigm, actions that include patients in their healthcare choices need to be encouraged. Shared decision making (SDM) is a collaborative mechanism that enables the clinician and patient to work together in making health decisions, exploring the alternatives, advantages, and negatives, while also recognizing the beliefs and perspectives of the patient [4]. To this end, there is a clear need for tools that support the subjective assessment and management of pain in a more quantifiable and objective way in order to improve care with a more effective approach.

Information and communication technologies (ICTs) are increasingly being used in pain care to address some of the challenges mentioned previously. Several topics are addressed through the use of ICTs, such as technology-enhanced pain treatment effectiveness/efficiency, pain management, technology-enhanced assessment or diagnosis of underlying conditions causing pain, etc. The widespread adoption of mobile health (mHealth) applications presents numerous advantages to all parties involved, leading to improved outcomes [5]. Although the utilization of mobile apps for pain management and tracking has been observed for several years, the initial endeavors were relatively rudimentary [6]. Preliminary investigations have highlighted the lack of consumer and clinician engagement and participation during the development process and the variations in app quality. Nowadays, an increasing number of apps are available for tracking, assessing, and managing pain [7,8], offering a wide range of features. However, the development of these apps for the wider market rarely follows scientific guidelines.

This systematic review makes significant contributions in several areas. The major findings and contributions of the review can be summarized as follows:

- Identification and synthesis of research attempts: The review examines the current state of progress in mHealth apps for pain management and identifies, interprets, and synthesizes the state-of-the-art research efforts. This includes investigating the design and assessment approaches, as well as the usability features reported in the scientific literature.
- Overview of available solutions in the market: The review provides an extensive overview of the existing mHealth apps for pain management that are available on the market. By analyzing these solutions, the review identifies gaps and areas for improvement in the field and serves as a guide for future pain app development.
- Comparison of research and commercial efforts: The review conducts a comparative analysis of the research and commercial endeavors in the field of mHealth apps for pain management. This comparison helps to reveal the key issues and challenges encountered in both domains and provides insights and recommendations for new endeavors.

## 2. Materials and Methods

The study included mobile apps designed for both patients and physicians; the app designs were focused on pain tracking, education, evaluation, and care. The systematic literature review (SLR) process and the preferred reporting items for systematic reviews and meta-analyses (PRISMA) guidelines were adopted [9]. Table 1 presents the defined research questions. The different phases of the research process are presented in the following pages.

### 2.1. Pain-Related Applications Reported on Scientific Databases

A search of peer-reviewed publications targeting pain-related mHealth apps was conducted over a four-month period (September 2019–January 2020). In the first stage, a collection of criteria and resources for the search terms were specified. Science Citation Index Expanded, Excerpta Medica database by Elsevier, Google Scholar, SCOPUS, Medline, and PubMed were the main sources. Other sources of data included the Directory of Open Access Journals, BMJ Journals, Wolters Kluwer—Ovid—Lippincott Williams & Wilkins, Health Reference Center Academic, Expert Reviews, Wolters Kluwer—Ovid, Wiley Online Library, Social Sciences Citation Index, SpringerLink Open Access, DiVA—Academic Archive Online, SciVerse ScienceDirect, Taylor & Francis Online—Journals, American Psychological Association, SpringerLink, Informa—Informa Healthcare, and references from relevant articles. As the scope of the subject is broad without a formal taxonomy, a number of appropriate key words were established to provide maximum coverage. According to PICO (patient/population, intervention, comparison, and outcomes) [10], the keywords used in the queries were as follows: (*ache OR pain) AND (mobile OR application OR app OR smartphone OR electronic OR PDA OR “Personal Digital Assistant” OR “hand-held device” OR mHealth OR ICT OR “Information and Communication Technologies” OR telemedicine OR telehealth OR tablet OR “virtual reality” OR “augmented reality”). The inclusion criteria are presented below:

- Papers after 2000 in English;
- Papers from peer-reviewed conferences/journals;
- Articles focused on mobile apps for pain management/assessment;
- Full or short versions of papers must be available (not abstracts);
- Studies involving mobile devices.

The systematic review paper applied exclusion criteria to ensure that the focus was on relevant and recent English-language research published in peer-reviewed sources. Excluded were papers published before the year 2000; non-English papers; those not published in peer-reviewed conferences or journals; articles unrelated to mobile apps for pain management or assessment; papers available solely as abstracts without full or short versions; and studies not involving the use of mobile devices. These criteria guaranteed the inclusion of comprehensive and applicable research while excluding abstract-only studies and those unrelated to mobile devices.

For quality evaluation purposes, the papers under review were assigned weights according to their importance. The criteria and their weights are set out in Supplementary Materials (Table S1), with a maximum score of 7 points. The research questions reported in Table 1a were supported by an appropriate data extraction process (Supplementary Materials, Table S2). The selected papers underwent a full-text review.

### 2.2. Commercially Available Pain-Related Applications

The data included basic details of the commercial pain apps that were available in the two major app stores. The included applications targeted patients and healthcare professionals for education, treatment, and assessment purposes related to pain. The search was conducted from December 2019 to June 2020. The main sources were the Google Play Store website [11], Vionza iTunes, the App Store search engine [12], and the myhealthapps website [13]. According to PICO, the keywords used in the queries were as follows: (Pain OR *ache) AND (management OR diary OR assessment OR education OR treatment OR track OR log OR record OR scale OR severity).

The inclusion criteria are presented below:

– Present in Play Store or App Store (or both);
– Details are given in description section;
– Category: Medical OR Health&Fitness OR Health&Wellness OR Education;
– Devices: Android smartphone, Tablets, iPhone, iPad.

The exclusion criteria for the systematic review were the following: apps that were not accessible in the two prominent app stores; apps lacking adequate details in the description section; apps falling outside the designated categories mentioned earlier; and apps incompatible with the specified devices. By employing these criteria, the systematic review aimed to concentrate on the apps readily available through the popular app stores that offered comprehensive information, aligned with the relevant categories, and were compatible with commonly utilized devices. This approach ensured a focused analysis on the most accessible and applicable apps within the review.

The apps under review were assigned weights according to their importance for quality evaluation purposes. Table S3 (Supplementary Materials) presents the criteria used and their weights, with a maximum score of 2 points.

The extracted features were transferred to a Microsoft Excel spreadsheet and then converted into a matrix format for the app review. The research questions reported in Table 1b were supported by an appropriate data extraction process (Supplementary Materials, Table S4).

The data were verified against the descriptions found on the app website or in related publications (if available). The abstracted metadata included: the app name, the developer, the developer’s country of residence, the language(s), the supported platform(s), the available versions (pro, free, lite, etc.) and price, the user rating, the pain type or the related condition, the app’s short description, the features, the category, the date and version of the last update, information on the design (healthcare professional—HCP—and/or patient involvement), and support information.

Sensitivity and complexity analyses were not necessary for the present investigation due to several reasons. First, the research question was straightforward and accompanied by precisely outlined criteria for including and excluding data. Second, the data exhibited a considerable level of homogeneity. Third, there were no significant uncertainties or conflicting pieces of evidence to address. Finally, we explicitly acknowledged the limitations of the study and the potential sources of bias.

## 3. Results

### 3.1. Scientific Publications

**Figure 1:**
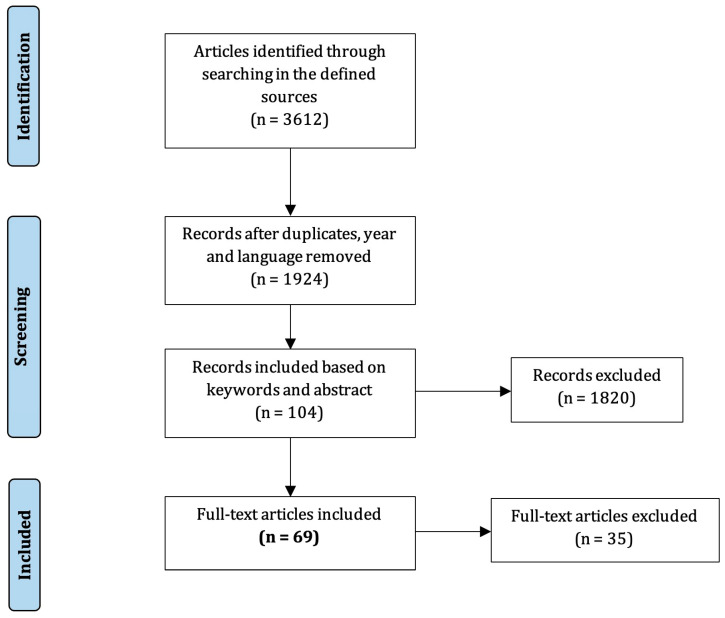
Flowchart of the search strategy and selection of articles.

**Figure 2:**
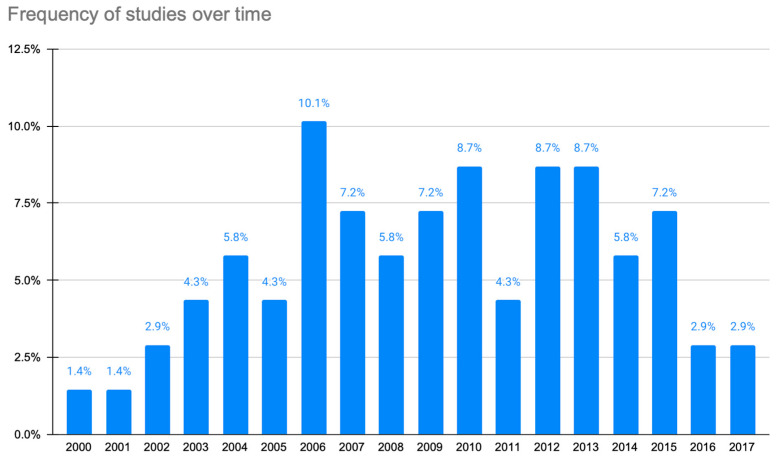
Chronological distribution of articles.

In total, 69 articles were identified and included in the study following the PRISMA guidelines (see Figure 1). The analytical results from the scientific publications are presented in Supplementary Materials (see Tables S5 and S6 [14,15,16,17,18,19,20,21,22,23,24,25,26,27,28,29,30,31,32,33,34,35,36,37,38,39,40,41,42,43,44,45,46,47,48,49,50,51,52,53,54,55,56,57,58,59,60,61,62,63,64,65,66,67,68,69,70,71,72,73,74,75,76,77,78,79,80,81]). Figure 2 illustrates the distribution of the research efforts over time.

**Figure 3:**
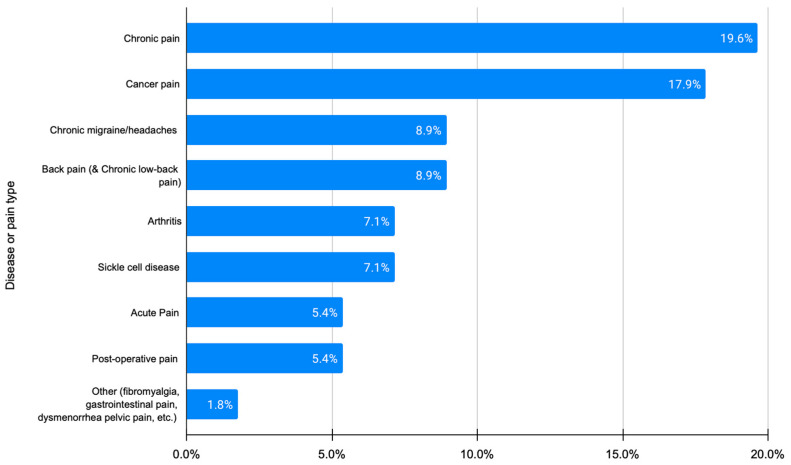
Target condition or pain type (for articles).

Almost all the apps (89.3%) were gender-neutral, while from an age perspective 60.7% targeted adults, followed by 30.4% that targeted children and adolescents (see Supplementary Materials—Figures S1 and S2). The data input methods used, which refer to standard pain measurement instruments and techniques, are briefly presented in Supplementary Materials (Table S7 and Figure S3). Regarding the types of pain assessment, two major categories are reported: static (i.e., at rest), at almost 41%, and dynamic (i.e., during some sort of activity), at almost 7%. These are also coupled with standard or customized questionnaires for data collection. The vast majority of the apps reported using an active mode for data input (98.2%) and only one app reported using a hybrid data input mode (active and passive through embedded sensors in device). The results are presented in Supplementary Materials (Figures S4 and S5). The targeted condition/pain types are presented in Figure 3.

**Figure 4:**
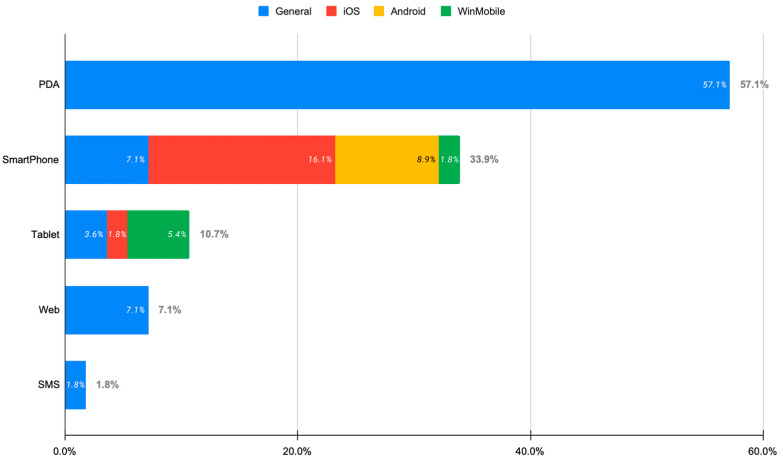
Technological approach followed by the identified apps (for articles).

Figure 4 depicts the technological approach used to deliver the reported service. This approach refers to both the target platform (iOS, Android, etc.) and the device used (smartphone, tablet, web, mobile phone, etc.). Regarding the subjective evaluation methods used, most of the apps reported using questionnaires and interviews (28.6% each), followed by focus groups and discussion (12.5%) and observation and field notes (8.9%) (see Supplementary Materials—Figure S6).

**Figure 5:**
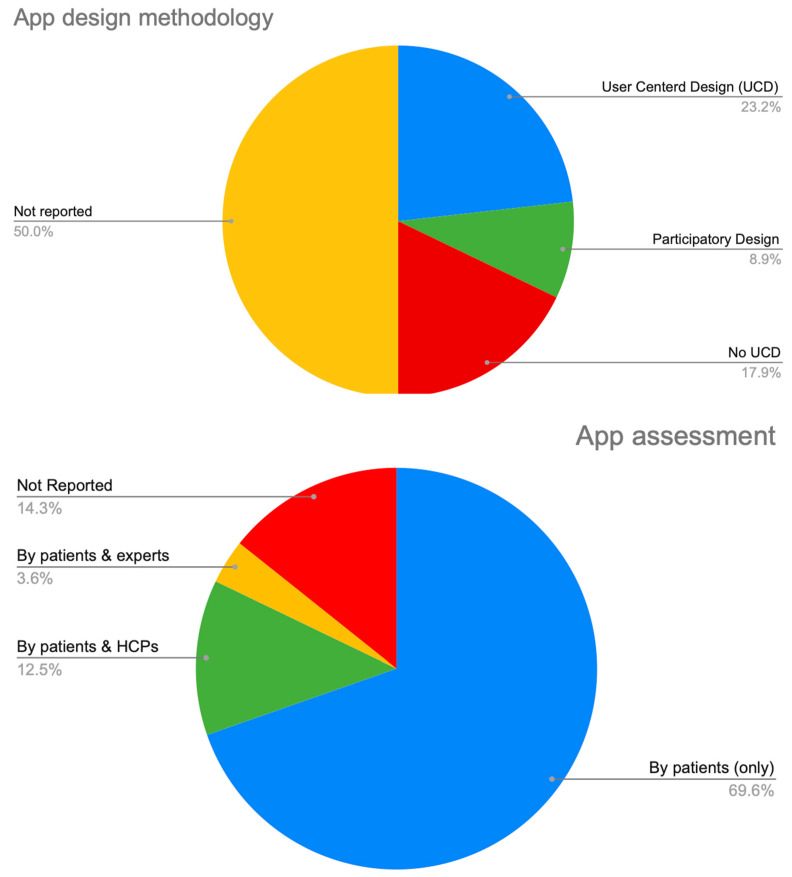
App design methodology and evaluation types (for articles).

Figure 5 presents the app design methodology and evaluation approaches.

### 3.2. Commercial Apps

**Figure 6:**
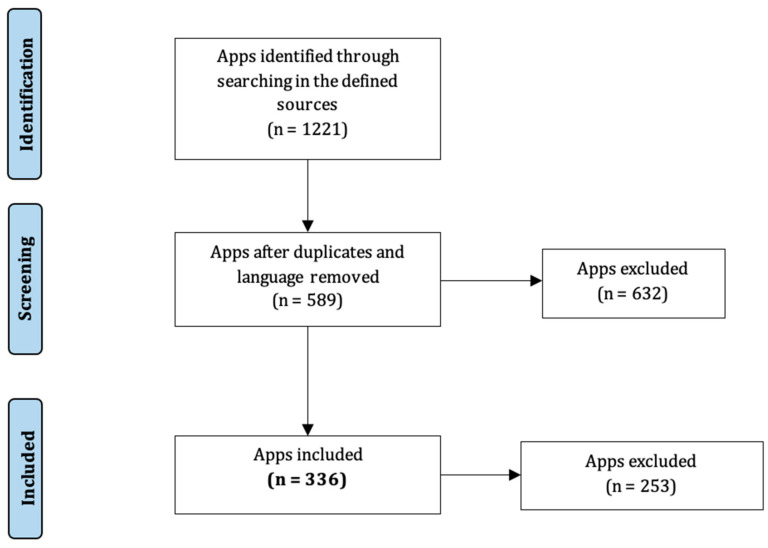
Flowchart of the search strategy and selection of commercial apps.

The inclusion requirements were met by a total of 336 apps (see Figure 6). The extracted data are presented in Supplementary Materials (Tables S8–S11). Almost 50% of them target only Android devices; 38% target iOS, while 11% target both platforms. General pain is the most common pain type supported (43.8%), followed by chronic migraine (26.5%) and back pain (13.1%). Regarding the design methodology, only 10.4% of the apps reported the participation of healthcare professionals, while patients’ participation was reported by only 2.1% of the apps. More detailed data are presented in Supplementary Materials (Figures S7–S9).

## 4. Discussion

According to the results, the majority (75.7%) of the scientific articles were published in medical journals. The wide distribution (with 41 different sources for 70 articles) could be interpreted as either the lack of a specialized source for the subject investigated in this study or that there were many reputed sources. The researchers seem to favor journals as the most “prestigious” and impactful sources when publishing their studies. Another significant finding is that only a small percentage of the published articles—7.1% (*n* = 5)—are in journals without a Journal Citation Reports (JCR) ranking, while all the conferences have a Conference Ranking by Computing Research and Education (CORE) ranking. This confirms that research in the field is a genuine need [5,82].

The quality assessment process revealed that almost all the scientific papers (90%) had a ranking exceeding half of the overall ranking. Six papers [44,64,66,76,78,79] ⁠scored 6; five [43,63,74,80,81]⁠ scored 5.5; and eight papers [14,35,42,50,53,70,73,75] scored 5. This indicates that for the conducted research, the reviewed papers were suitable.

All the papers were published from 2000 onwards, with the majority of them (70%) being published after 2006, as expected. Even though smartphones underwent substantial growth from 2012 [83,84,85], mHealth research efforts had already started in 2008 [86]. It is anticipated that the appearance of new and cheaper sensors and wearables will further push the research activities in this direction. Until 2012, most of the efforts were based on personal digital assistant (PDA) devices. Even though almost one-third of the research articles appeared after 2012, one could expect a much larger number of efforts in this period, when the smartphone market was already mature. This, compared to the broader research efforts in the field of mobile health [5,82], highlights that the field of pain had not yet attracted the interest of researchers, at least from a technological point of view.

The research efforts were mostly focused on the iOS operating system. This is interesting since Android is an open-source platform which controls the mobile operating systems (OS) market with almost 73% of the worldwide market share. This can be justified by the fact that iOS was the first operating system in smartphones which dominated the market at its early stage, with numerous characteristics: stability, user friendliness, user acceptance, etc. Moreover, Android devices suffer from heterogeneity with regard to OS versions (Android custom ROMs) and hardware features. On the other hand, half of the commercial apps identified run on Android OS and 38% run on iOS, which aligns with the commercial trend in the mobile market. Regarding the devices, tablets account for only 10.7% of the research efforts. It should be considered that the lifestyle of the contemporary citizen nowadays demands portable, non-intrusive, and ubiquitous solutions. The smartphone is the only device always available during a person’s daily activities. That is why all recent efforts have targeted smartphones. The above analysis reveals that new efforts should target both operating platforms. It is also evident that artificial intelligence, big data, and cloud computing were not exploited as expected. An open data philosophy could further support the emergence of novel solutions. Security concerns should be handled very carefully and in accordance with the relevant European Union laws and directives, such as the General Data Protection Regulation [87], Directive 95/46/EC [88], Directive 2002/58/EC [89], and the Charter of Fundamental Rights [90].

The research findings reveal the absence of automatic pain data recording and the challenges in objective pain measurement, while highlighting the need for dedicated wearable pain sensors. Existing smartphone sensors (accelerometer, gyroscope, heart rate, etc.) are inadequate for this purpose. Developing sensors combined with artificial intelligence techniques is crucial. Although self-reports are convenient, they have limitations (they are subjective and inconsistent, they cannot be obtained reliably from mentally impaired persons, etc.). Recent research aims to leverage existing or new sensors for objective pain assessment, integrating data from various physiological signals [91,92,93]. Most of these efforts implement algorithms and artificial intelligence techniques to combine data that are mainly related to heart rate variability, skin conductivity, blood pressure, tension in face muscles, eye movements, and brain signals. However, no similar endeavors were found in the reviewed literature and commercial apps. The integration of such sensors could boost efforts in the examined domain. This could help towards a generic input method for pain, which now appears to be scattered as it is closely related to the underlying health condition (see Supplementary Materials—Table S7).

Another significant finding is that half of the research efforts did not report any design methodology, while only 23.2% of them followed user-centered design (UCD), 8.9% followed participatory design, and 17.9% reported that they did not follow UCD. This is consistent with the latest data: a significant number (50,000) of monthly active users (MAUs) were reported by only 7% of mHealth apps [94], while the vast majority of mHealth apps (62%) reported fewer than 1000 MAUs. According to previous research, the average time it takes for an mHealth app to be uninstalled after the last usage session is 8.8 days [95]. Global statistics indicate that mHealth apps exhibit one of the highest uninstall rates, reaching 27.8% [96]. Several factors contribute to this high uninstall rate, including:

- Hardware issues: Problems such as battery drain and device incompatibility.
- First impression: The likelihood of uninstallation is greater during the initial day of app usage.
- Poor user experience: Issues such as excessive bugs, confusing user interface, unattractive aesthetics, overwhelming features, and unfulfilled expectations.
- Steep learning curve: Users find it time-consuming and difficult to learn how to navigate the app.
- Lack of value and desired features: Insufficient usefulness and absence of features that align with users’ daily needs.
- Privacy concerns: Too many permission requests without an explanation why.
- Poor engagement: Too many notifications that annoy users or too little communication, which makes them forget the app.
- Content: The content is not updated regularly with evidence-based data due to the limited participation of HCPs.

The above data reveal that a co-design approach is a key factor towards mHealth success.

Another critical issue is the subjective assessment of the app to ensure, at an early stage, that it meets the expectations of its end users. This task is very challenging and time-consuming. However, to date, there is no standard assessment methodology to follow. Regarding the research efforts, almost half of them (57%) did not report any subjective evaluation. Usability evaluation was exclusively performed by real end users (patients) in 69.6% of cases, while HCPs and other experts participated in 12.5% and 3.6%, respectively. Finally, 14.3% did not report any usability evaluation at all. The absence of a standardized evaluation framework significantly impacts the design and sustainable growth of mHealth apps and hinders their long-term adoption. Many commonly used usability scales were initially developed for websites or computer software and do not specifically cater to app-focused assessments. Examples of such scales include the perceived Usefulness and Ease of Use (PUEU) questionnaire [97], the Software Usability Measurement Inventory (SUMI) [98], and the System Usability Scale (SUS) [99]. Consequently, evaluating the “quality” of mHealth apps proves to be a challenging task. In recent years, efforts have been made to develop scales specifically tailored to the rating of mHealth apps, albeit with a focus on specific health domains. Notable examples include the APPLICATIONS scoring system for pregnancy apps [100], the National Institute of Health and Care Excellence (NICE) for behavior change apps [101], the MedAd-AppQ for medical adherence apps [52,102], the Nutrition App Quality Evaluation for nutrition apps [103], and the Quality Assessment Tool for Evaluating Medical Apps for medication complication apps [104]. However, the reliability and validity of most of these scales are yet to be substantiated with empirical evidence. Several general scales have emerged for the purpose of assessing the quality of mHealth apps, such as the Health Care Apps Evaluation Tool [105], the Organisation for the Review of Care and Health Applications—24 Question Assessment [106], and the Mobile Application Rating Scale (MARS) [107]. Only MARS has been developed to be used by the general public. The limited scope of the aforementioned scales makes it hard to find the right tool to evaluate the quality of an mHealth app targeting the pain domain. Moreover, most scales do not cover all aspects of quality, such as price and value. The heterogeneity in the assessment criteria in the rating scales is another issue for the researchers and developers. We can conclude that there is still a great need to establish a credible and effective method for subjective evaluation purposes [108].

Another problem in the mHealth domain is that, to date, there is no guidance for the end users (patients and healthcare professionals) to find the app that matches their needs. However, recently, several regulations have appeared that can be applied to mHealth apps as well [109,110,111]. These regulations, such as Medical Device Reporting (MDR), and/or those of the US Food and Drug Administration (FDA) are a step forward towards supporting end users in choosing the appropriate application. Therefore, it should be considered obligatory for developers and researchers to follow them. In our research, none of the identified applications were MDR- and/or FDA-certified.

In conclusion, to optimize the overall quality and retention rate of an mHealth app it is important to ensure:

- Active participatory design, involving different end users and stakeholders (HCPs, patients, and healthcare organizations).
- Appropriate subjective assessment.
- Optimal user experience.
- Communication of the benefits of the app and what the end user is missing out on by not using the app.

In terms of the targeted pain type, the commercial apps are not aligned with the research efforts, as shown in Figure 3 and Figure S8 (Supplementary Materials). More specifically, almost half of the commercial apps (43.8%) focus on general pain, 26.5% on migraine/headache (instead of 8.8% in the research efforts), 13.1% on back pain (instead of 8.8%), and 3.3% on arthritis (instead of 7%). Based on the above, commercial apps seem to focus on the most prevalent conditions, as expected, in order to reach a larger audience. For example, migraine is the third most prevalent illness in the world, while back pain represents 27% of all pain types [112]. The fact that only a minimal number of commercial apps reported the involvement of either patients (2%) or HCPs (10%) in the design and development phases also reveals their commercial and technical focus.

The majority (60.7%) of the research efforts targeted adults. Nevertheless, there is no evidence of research apps specifically focusing on the elderly. The ageing of the population, combined with the latest findings that pain significantly increases with age [113], urges innovative solutions explicitly focusing on this group. It is therefore essential for researchers and developers to design mHealth solutions that take into account the digital literacy or technophobia issues of the end users, as well as other problems (low cognitive level, vision problems, physical impairments, etc.).

### Research Limitations

For scientific studies, one possible limitation is that the scope was mainly targeted at articles published until the end of 2019. As for the commercially available apps, the main limitations of this study include:

- Country and language: English apps in App Store and Play Store.
- Search facilities (especially for app stores): Using a third-party service (such as Vionza) does not guarantee that we receive the same results as those of the official stores.
- We acknowledge the significance of incorporating more recent data and are already working to expand our research in future endeavors to encompass the latest developments, ensuring the timeliness and relevance of our findings. However, we believe that the period we examined is crucial, particularly due to the emergence of COVID-19, which significantly accelerated the proliferation of mHealth apps across various domains. Hence, we consider this timeframe as a distinct era that warrants separate investigation.

## 5. Conclusions

In conclusion, this study aimed to review the current trends in both the commercial and research domains related to pain-focused apps, with a focus on identifying the key issues and guidelines for the effective development of these apps. Through a systematic review of scientific publications and commercially available apps, the study revealed several critical points that should be considered in future efforts.

The analysis of the features of the included articles and apps highlighted important findings. Firstly, it was observed that commercial and research efforts often target different types of pain, indicating the need for a more comprehensive approach. Additionally, the majority of the examined apps lacked participatory design, suggesting a lack of user involvement in the development process. Furthermore, evaluating the final apps remained a challenge, potentially hindering their success.

The study also emphasized the importance of patient empowerment and shared decision making in the pain domain. The subjective nature of pain necessitates tools that can facilitate pain assessment and management in a more objective and quantifiable way. ICTs, particularly mHealth applications, have the potential to address these challenges and improve outcomes. However, the development of pain-focused apps for the wider market often falls short in the following of scientific guidelines.

Looking ahead, the study suggests that the pain domain is expected to experience a substantial increase in the development of non-intrusive wearables and sensors for pain detection and assessment, along with the integration of artificial intelligence techniques and open data. More research is needed to bridge the gap between commercial and research efforts, enhance user engagement in the development process, and improve the evaluation of pain-focused apps.

In summary, this study provides valuable insights into the current landscape of pain-focused apps, highlighting areas for improvement and offering recommendations for future endeavors. By addressing the identified challenges and leveraging the potential of ICTs, it is possible to develop more effective tools for pain assessment, management, and shared decision making and ultimately to improve the quality of care for individuals experiencing chronic pain.

## Figures and Tables

**Table 1:** (**a**) Research questions (for articles); (**b**) research questions (for commercial apps).

(**a**)		
**No.**	**Research Question**	**Objective**
RQ1	What are the target groups?	To classify (number, characteristics, and types) the population targeted (patients, healthcare professionals, and carers).
RQ2	What health conditions are targeted?	To describe the particular symptoms and health problems that are examined.
RQ3	What is the technological approach followed?	To report data on the various technological approaches in terms of hardware, software, sensors, artificial intelligence, etc.
RQ4	What is the chronological distribution of the publications?	To report the frequency of research on pain apps over time.
RQ5	What assessment methodologies are used?	To analyze the methodologies of evaluation (objective or subjective) of the applications.
RQ6	What methodology is followed for pain assessment?	To analyze the methodologies of pain assessment (objective or subjective).
RQ7	Which methods are used to assess user acceptance?	To examine the user friendliness, user acceptance, and human–computer interaction (HCI) methodologies.
(**b**)		
**No.**	**Research Question**	**Objective**
RQ8	What health conditions are targeted?	To classify (number, characteristics, and types) the population targeted (patients, healthcare professionals, and carers).
RQ9	What is the targeted platform of the app?	To provide information on the targeted platforms of the selected apps.
RQ10	Design methodology	To report end users’ involvement in the design process.

## Data Availability

The data presented in this study are available in Supplementary Materials.

## Supplementary Materials

The following supporting information can be downloaded at: https://www.mdpi.com/article/10.3390/s23156965/s1, Table S1: Quality assessment criteria (for articles), Table S2: Data extraction (for articles), Table S3: Quality assessment criteria (for commercial apps), Table S4: Data extraction (for commercial apps), Table S5: Selected papers and review results #1, Table S6: Selected papers and review results #2, Table S7: Pain measurement instruments and techniques, Table S8: Pain apps available in App Store for iOS (Vionza), Table S9: Pain apps available in Google Play for Android, Table S10: Pain apps reported in myhealthapps.net, Table S11: Commercial Apps data table, Figure S1: Target population (age) for selected articles, Figure S2: Target population (gender) for selected articles, Figure S3: Data input method for pain assessment (articles), Figure S4: Types of pain assessment (articles), Figure S5: Types of data input mode (articles), Figure S6: Subjective evaluation methods (articles), Figure S7: Target platform for commercial pain management apps, Figure S8: Target condition or pain type for commercial pain management apps, Figure S9: Design methodology for commercial pain management apps.
