# Supplementary material for: Pain Management Mobile Applications: A Systematic Review of Commercial and Research Efforts
Source: Sensors (Basel). 2023 Aug 5;23(15):6965. doi: 10.3390/s23156965 (PMC10422642; doi:10.3390/s23156965)
Supplement: Supplementary file 1 [file sensors-23-06965-s001.zip › Appendix A.pdf]

No.	Quality assessment question	Weight
QC1	Targeted condition specified?	Yes (+1), No (+0)
QC2	Targeted population specified?	Yes (+1), No (+0)
QC3	Is the technological solution presented in details?	Yes (+1), Partially (+0.5), No (+0)
QC4	Is the pain assessment methodology presented in details?	Yes (+1), Partially (+0.5), No (+0)
QC5	Is the evaluation/assessment method specified and presented in details?	Yes (+1) / Partially (+0.5), No (+0)
QC6	Is the paper published in a recognized source?	Conferences, Workshops: CORE A* or A (+1.5), CORE B (+1), CORE C (+0.5), not included in CORE ranking (+0).<br>Journals:<br>ranked Q1 (+2), ranked Q2 (+1.5), ranked Q3 or Q4 (+1), no JCR ranking (+0)<br>Other sources: (+0)<br>Books, Lecture Notes: (+2)

Table S1. Quality assessment criteria (for articles)

No.	Extracted data
RQ1	Categorization of the population targeted should be defined (e.g., patients, carers, teachers, etc.).
RQ2	The health topic should be extracted to identify the health condition addressed.
RQ3	The technological domain addressed should be presented (e.g., hardware or software solution, other related info like the use of artificial intelligence, etc.).
RQ4	Publication year is required.
RQ5	The technology assessment methodology should be presented.
RQ6	The pain assessment methodology should be presented.
RQ7	User assessment methodology should be presented

Table S2. Data extraction (for articles)

No.	Quality assessment question	Weight
QC1	Targeted condition specified?	Yes (+1), No (+0)
QC2	Design methodology described	Yes (+1), No (+0)

Table S3. Quality assessment criteria (for commercial apps)

No.	Extracted data
RQ1b	The health topic should be extracted to identify the health condition addressed.
RQ2b	The targeted platform should be presented
RQ3b	The design methodology should be presented (e.g., involvement of patients and HCPs).

Table S4. Data extraction (for commercial apps)

Ref.	QC1	QC2	QC3	QC4	QC5	QC6	QA Score
Peters et al., 2000 [14]	1.0	1.0	0	0.5	0.5	2.0	5.0
Jamison et al., 2001 [15]	1.0	1.0	0	0	0	2.0	4.0
Jamison et al., 2002 [16]	1.0	1.0	0	0	0.5	2	4.5
Walker et al., 2002 [17]	1.0	1.0	0	0.5	0.5	1.5	4.5
Goldstein et al., 2003 [18]	1.0	1.0	0	0	0	1.5	3.5
Stone et al., 2003 [19]	1.0	1.0	0	0	0	2.0	4.0
VanDenKerkhof et al., 2003 [20]	1.0	1.0	0	0	0	1.5	3.5
Chan et al., 2004 [21]	1.0	1.0	0	0	0	1.0	3.0
Gaertner et al., 2004 [22]	1.0	1.0	0	0	0	2.0	4.0
Palermo et al., 2004 [23]	1.0	1.0	0	0	0.5	2.0	4.5
Roelofs et al., 2004 [24]	1.0	1.0	0	0	0	2.0	4.0
Serif and Ghinea, 2005 [25]	1.0	1.0	0	0	1.0	0	3.0
Aaron et al., 2005 [26]	1.0	1.0	0	0.5	0	2.0	4.5
Turner et al., 2005 [27]	1.0	1.0	0	0.5	0	2.0	4.5
Kearney et. al., 2006 [28]	1.0	1.0	0	0	1.0	1.5	4.5
Aaron et al., 2006 [29]	1.0	1.0	0	0.5	0	0	2.5
Jamison et al., 2006 [30]	1.0	1.0	0	0	0	2.0	4.0
Roelofs et al., 2006 [31]	1.0	1.0	0	0	0	1.5	3.5
Sorbi et al., 2006 [32]	1.0	1.0	0	0	0	1.5	3.5
Sorbi et al., 2006 [33]	1.0	1.0	0	0	0	1.5	3.5
Stinson et al., 2006 [34]	1.0	1.0	0	0.5	0.5	1.5	4.5
Sorbi et.al., 2007 [35]	1.0	1.0	0	0	1.0	2.0	5.0
Evans et al., 2007 [36]	1.0	1.0	0	0.5	0	1.5	4.0
Goldberg et al., 2007 [37]	1.0	1.0	0	0.5	0	2.0	4.5
Heiberg et al., 2007 [38]	1.0	1.0	0	0.5	0	2.0	4.5
Ghinea et al., 2008 [39]	1.0	1.0	0	0.5	0	0	2.5
Junker et al., 2008 [40]	1.0	1.0	0	0	0	1.5	3.5
Stinson et al., 2008 [41]	1.0	1.0	0	0.5	0.5	1.0	4.0
Stinson et al., 2008 [42]	1.0	1.0	0	0.5	0.5	2.0	5.0
Anatchkova, et. al., 2009 [43]	1.0	1.0	0	0.5	1.0	2.0	5.5
McClellan et al., 2009 [44]	1.0	1.0	1.0	0.5	1.0	1.5	6.0
Gulur et al., 2009 [45]	1.0	1.0	0	0	0.5	2.0	4.5
Lewandowski et al., 2009 [46]	1.0	1.0	0	0	0.5	1.5	4.0
Kleiboer et al., 2009 [47]	1.0	1.0	0	0	0.5	1.0	3.5
Sorbi et al., 2010 [48]	1.0	1.0	0	0	0.5	2.0	4.5
Hachizuka et al., 2010 [49]	1.0	1.0	0.5	0	1.0	1.0	4.5

Luckmann and Vidal, 2010 [50]	1.0	1.0	0.5	0	1.0	1.5	5.0
Alfvén 2010 [51]	1.0	1.0	0	0.5	0.0	1.5	4.0
Connelly et al., 2010 [52]	1.0	1.0	0	0.5	0.0	2.0	4.5
Connelly et al., 2010 [53]	1.0	1.0	0.5	0.5	0	2.0	5.0
Marceau et al., 2010 [54]	1.0	1.0	0	0.5	0.0	2.0	4.5
Connelly et al., 2011 [55]	1.0	1.0	0.5	0.5	0	1.5	4.5
Kristjánsdóttir et al., 2011 [56]	1.0	1.0	0.5	0	0	1.0	3.5
Rosser et al., 2011 [57]	1.0	1.0	0	0	0	1.0	3.0
Rosser and Eccleston, 2011 [6]	1.0	1.0	0	0	0	1.0	3.0
Wood et al., 2011 [58]	1.0	1.0	0	0.5	0	1.5	4.0
Allena et al., 2012 [59]	1.0	1.0	0	0.5	0	2.0	4.5
Baggott et al., 2012 [60]	1.0	1.0	0	0	1.0	0	3.0
Jibb et al., 2012 [61]	1.0	1.0	0	0	0	2.0	4.0
Spyridonis et al., 2012 [62]	1.0	1.0	0	0	1.0	0.5	3.0
Jacob et al., 2012 [63]	1.0	1.0	1.0	0.5	1.0	1.0	5.5
Jacob et al., 2013 [64]	1.0	1.0	1.0	0.5	1.0	1.5	6.0
Nes et al., 2013 [65]	1.0	1.0	0.5	0	0	2.0	4.5
Stinson et al., 2013 [66]	1.0	1.0	1.0	0	1.0	2.0	6.0
Kristjánsdóttir et al., 2013 [67]	1.0	1.0	0.5	0	0	2.0	4.5
Kristjánsdóttir et al., 2013 [68]	1.0	1.0	0.5	0	0	2.0	4.5
Blödt et al., 2014 [69]	1.0	1.0	0.5	0	0	1.0	3.5
Garcia-Palacios et al., 2014 [70]	1.0	1.0	0.5	0	1.0	1.5	5.0
Jibb et al., 2014 [71]	1.0	1.0	0.5	0	1.0	0	3.5
Pombo et al., 2014 [72]	1.0	1.0	0.5	0	1.0	1.0	4.5
Bakshi et al., 2015 [73]	1.0	1.0	0.5	0	1.0	1.5	5.0
Huguet et al., 2015 [74]	1.0	1.0	0.5	0	1.0	2.0	5.5
Jonassaint et al., 2015 [75]	1.0	1.0	1.0	0	1.0	1.0	5.0
Maguire et al., 2015 [76]	1.0	1.0	1.0	0	1.0	2.0	6.0
Nguyen et al., 2015 [77]	1.0	1.0	1.0	0	1.0	0.5	4.5
Fortier et al., 2016 [78]	1.0	1.0	1.0	0	1.0	2.0	6.0
Hochstenbach et al., 2016 [79]	1.0	1.0	1.0	0	1.0	2.0	6.0
Ingadottir et al., 2017 [80]	1.0	1.0	1.0	0	1.0	1.5	5.5
Jibb et al., 2017 [81]	1.0	1.0	1.0	0	1.0	1.5	5.5

Table S5. Selected papers and review results #1

Ref.	Pain Type	Targeted population	Smart device	Pain assessment method	App assessment
Peters et al., 2000 [14]	Unexplained pain	Adults	PDA	Not reported	Not reported
Jamison et al., 2002 [16]	Healthy volunteers	Adults	PDA	Not reported	Not reported
Walker et al.; 2002 [17]	Gastrointestinal pain	Children (6–10 years old)	PDA	Not reported	Patients
Goldstein et al., 2003 [18]	Post-operative pain, Hernia	Adults	PDA	Not reported	Not reported
Stone et al., 2003 [19]	Chronic Pain	Adults	PDA	Not reported	Patients
VanDenKerkhof et al., 2003 [20]	Acute Pain	Adults	PDA	Not reported	Patients
Chan et al., 2004 [21]	Acute Pain	Adults	PDA	Not reported	Patients
Gaertner et al., 2004 [22]	Cancer pain	Adults	PDA	Not reported	Patients
Palermo et al., 2004 [23]	Headaches and Juvenile Idiopathic Arthritis	Children and adolescents (8–16 years old)	PDA	Not reported	Patients
Serif and Ghinea, 2005 [25]	Chronic back pain	27–65 years, both male and female, literacy level not mentioned	PDA	Dynamic & Standard	Patients/HCPs (questionnaires)
Kearney et. al., 2006 [28]	Cancer pain	24–77 years, both male and female	handheld computer	Static & Standard	Patients/HCPs (questionnaire, interview, software log of activity)
Aaron et al., 2005 [26], Aaron et al., 2006 [26], Turner et al., 2005 [27]	Chronic temporo-mandibular pain	Adults	PDA	Not reported	Not reported
Jamison et al., 2001 [15], Jamison et al., 2006 [30]	Chronic low-back pain	Adults	PDA	Not reported	Not reported

Roelofs et al., 2004 [24], Roelofs et al., 2006 [31]	Chronic low-back pain	Adults	PDA	Not reported	Patients
Sorbi et al., 2006 [32], Sorbi et al., 2006 [33]	Chronic pain	Adults	PDA	Not reported	Patients
Sorbi et.al., 2007 [35], Sorbi et al., 2010 [48]	Chronic migraine	34-52 years females	PDA	Static & Standard	Patients (questionnaire and interview)
Evans et al., 2007 [36]	HIV-associated sensory neuropathies (HIV-SN)	Adults	PDA	Not reported	Patients
Goldberg et al., 2007 [37]	Menstrually related headache	Adult females	PDA	Not reported	Patients
Heiberg et al., 2007 [38]	Rheumatoid arthritis	Adults	PDA	Not reported	Patients
Ghinea et al., 2008 [39]	Back pain	Adults	PDA	Not reported	Patients/HCPs
Junker et al., 2008 [40]	Chronic pain	Adults	PDA	Not reported	Not reported
Stinson et al., 2006 [34], Stinson et al., 2008 [41], Stinson et al., 2008 [42]	Chronic pain of juvenile idiopathic arthritis	Children and adolescents 9-18 years, both male and female	PDA	Dynamic & Standard	Patients (think aloud, interview)
Anatchkova, et. al., 2009 [43]	Chronic Pain	>=18 years, both male and female	Tablet PC	Static & Standard	Patients (questionnaire)
McClellan et al., 2009 [44]	Chronic vaso-occlusive pain (sickle cell disease)	Children and adolescents (8–20 years old), both male and female	PDA	Static & Standard	Patients / Care takers (questionnaire)
Gulur et al., 2009 [45]	Acute pain	Children and adolescents (3–17 years old)	PDA	Not reported	Patients
Lewandowski et al., 2009 [46]	Chronic pain	Children and adolescents (8–16 years old)	PDA	Not reported	Patients
Kleiboer et al., 2009 [47]	Migraine headache	Adult females	PDA	Not reported	Patients

Hachizuka et al., 2010 [49]	Cancer pain	>=20 years, both males and females	PDA	Static & Standard	Patients (interview)
Luckmann and Vidal, 2010 [50]	Non-cancer chronic pain	not specified	PDA	Static & Standard	Patients (notes, discussion)
Alfvén 2010 [51]	Chronic pain	Children and adolescents (9–15 years old)	SMS	Not reported	Patients
Connelly et al., 2010 [52]	Headaches	Children and adolescents (8–17 years old)	PDA	Not reported	Patients
Marceau et al., 2010 [54]	Non-cancer chronic pain	Adults	PDA	Not reported	Patients/HCPs
Rosser et al., 2011 [57], Rosser and Eccleston 2011 [6]	Chronic pain	Both male and female	Smartphone	Static & Standard	Not reported
Wood et al., 2011 [58]	Postoperative disease-related pain	Children (4–12 years old)	PDA	Not reported	Patients
Baggott et al., 2012 [60]	Cancer pain	13-21 years, both male and female	Smartphone (iOS)	Static & Standard	Patients (questionnaire, think-aloud and interview)
Spyridonis et al., 2012 [62]	Musculoskeletal pain (body pain in wheelchair patients)	>=18 years, both male and female	Smartphone and tablets (android)	Static & Standard	Patients (questionnaire)
Allena et al., 2012 [59]	Headaches	Adults	PDA	Not reported	Patients
Connelly et al., 2010 [53], Connelly et al., 2011 [55]	Juvenile Idiopathic Arthritis	Adolescents (8–18 years old)	Smartphone	Not reported	Patients
Jacob et al., 2012 [63], Jacob et al., 2013 [64]	Chronic pain in sickle cell disease	Children and adolescents (10–17 years old), both male and female	Smartphone	Static & Standard	Patients
Nes et al., 2013 [65]	Chronic widespread musculoskeletal pain	Female, age not specified	PDA / Smartphone	Static & Standard	Patients (questionnaire, interview)

Stinson et al., 2013 [66]	Cancer pain	9-18 years, both male and female	Smartphone (iOS)	Static & partially customized	Patients (interviews, questionnaire)
Jibb et al., 2012 [61]	Cancer pain	Children and adolescents (8–18 years old)	Smartphone (iOS)	Not reported	Patients
Kristjánsdóttir et al., 2011 [56], Kristjánsdóttir et al., 2013 [67], Kristjánsdóttir et al., 2013 [68]	Chronic widespread pain	Adults	Smartphone	Not reported	Patients
Blödt et al., 2014 [69]	Chronic Low Back Pain - Chronic Neck Pain	18 to 65 years, gender not specified	Smartphone	Static & Standard	Not reported
Garcia-Palacios et al., 2014 [70]	Chronic pain in fibromyalgia	37-65 years, female	Smartphone (Win mobile)	Static & Customized	Patients (questionnaire)
Jibb et al., 2014 [71]	Pediatric cancer pain	12-18 years, both male and female	Smartphone (iOS)	Static & Standard	HCPs / adolescents (interviews)
Pombo et al., 2014 [72]	Acute post- operative pain	18–75 years, both male and female	Smartphone	Static & Standard	Patients (questionnaire)
Bakshi et al., 2015 [73]	Vaso- occlusive pain - sickle cell disease	Adolescents and Young Adults, both male and female	Smartphone	Static & Standard	Experts/Patients (observation & field notes, interview)
Huguet et al., 2015 [74]	Headache	14-28 years, both male and female	Smartphone (iOS)	Static & Standard	Patients (questionnaire, interview)
Jonassaint et al., 2015 [75]	Chronic vaso- occlusive pain (sickle cell disease)	16–54 years, both male and female	Smartphone – Tablet (iOS)	Dynamic & Customized	Patients (interview)
Maguire et al., 2015 [76]	Lung Cancer Pain	>=18 years, both male and female	Smartphone	Static & Customized	Patients / HCPs (interview, questionnaire)
Nguyen et al., 2015 [77]	Dysmenorrhea pelvic pain	14–50 years, female	PDA	Static & Standard	Patients (think-aloud, interviews)

Fortier et al., 2016 [78]	Cancer pain	8-18 years, both male and female	TabletPC (Android)	Static & Standard	Patients (questionnaire)
Hochstenbach et al., 2016 [79]	Cancer pain	Age not specified, both male and female	Tablet (iOS)	Static & Standard	Experts/HCPs/Patients
Ingadottir et al., 2017 [80]	Post- operative pain	>=18 years, both male and female	TabletPC (Android)	Static & Standard	Patients (questionnaire, interview, focus group, discussion)
Jibb et al., 2017 [81]	Cancer pain	12-18 years, both male and female	Smartphone (iOS)	Dynamic & Standard	Patients (observation, think-aloud, interview)

Table S6. Selected papers and review results #2

Method	Description	Percentage
ASK	Activity Scale for Kids	2%
CEMS	Children's Emotion Management Scale	2%
CES-D	Center for Epidemiological Studies - Depression Scale	2%
CSQ	Coping Strategies Questionnaire	2%
FBP-RS	Faced Based Pictorial - Rating Scale	2%
FPS / FPS-R	Faces Pain Scale / Faces Pain Scale – Revised	4%
GPS	Gracely Pain Scale	2%
LIKERT	LIKERT scale	2%
LO	List of Options	9%
MCQs	Multiple Scale Questions	21%
mHAQ	modified Health Assessment Questionnaire	2%
MIDOS	MInimal DOcumentation System	2%
MPI	Multidimensional Pain Inventory	2%
NRS	Numeric Rating Scale	16%
OA	Open Answers	13%
PAD	Pain And Distress ratings	2%
PANAS-C	Positive And Negative Affect Scale - for Children	4%
PCS	Pain Catastrophizing Scale	2%
PD	Pain Drawing	20%
PD-Q	Pain DETECT - Questionnaire	2%
SPS	Standard Pain Scoring	23%
VAS	Visual Analog Scale	27%
VRS	Verbal Rating Scale	2%

Table S7. Pain measurement instruments and techniques

App Name	Developer	Price	Category	Store	Devices	Pain problem (condition)
AccuRelief 3-in-1 Pain Relief	Compass Health Brands	Free	Medical	US	iPhone	Pain
ACPA Pain Logs	The American Chronic Pain Association, Inc.	Free	Health & Fitness	US, UK, CA	iPhone, iPad	Pain
Back to Health		Free	Health & Fitness	UK	iPhone, iPad	Back pain
Chronic Pain Diary	Ben Delaporte	Free	Medical	US, UK, CA	iPhone	Pain
Chronic Pain Diary Lite	Ben Delaporte	Free	Medical	US, UK, CA	iPhone	Pain
Chronic Pain Tracker	Chronic Stimulation, LLC	$6,99/Lite: free	Medical	US, UK, CA	iPad, iPhone	Pain
EPM - Essential Pain Management	Qworks Technologies Pvt Ltd	Free	Medical	US, UK	iPhone	Pain
HeadacheDiary - Lite	Tim Preuss	Free	Health & Fitness	UK, CA	iPhone	Headache
iBeatPain for Teens	Take The Wind, Lda.	Free	Health & Fitness	US	iPhone	Pain
iMigraine - migraine tracker	Softarch Technologies AS	Free	Medical	US, UK, CA	iPad, iPhone	Migraine
iPain	Anouk Stein, M.D.	Free	Medical	CA	iPhone	Pain
Manage My Pain	ManagingLife	Free	Medical	US, UK, CA	iPad, iPhone	Pain
Migraine Buddy	Healint	Free	Medical	US, UK, CA	iPad, iPhone	Migraine
My Pain Diary & Symptom Tracker: Gold Edition	Damon Lynn	$ 4,99	Medical	US,UK, CA	iPad, iPhone	Allergies, back pain, chronic fatigue syndrome (CFS), depression, fibromyalgia,

						headaches, or migraine
My Pain Diary: Chronic Pain & Symptom Tracker	Damon Lynn	$ 4,99	Medical	US,UK, CA	iPhone	Pain
My Pain Log	Samantha Roobol	Free	Medical	US, UK, CA	iPad, iPhone	Pain
My Pain Logs	Subinprara Infotech Inc.	Free	Medical	US, UK, CA	iPad, iPhone	Pain
Pain App	University of Greenwich	Free	Medical	US, UK	iPad, iPhone	Pain
Pain Care App	appliedVR	Free	Health & Fitness	US	iPhone, iPad	Pain
Pain Management Plan	Advanced Digital Institute - Health	free	Medical	UK	iPhone	Pain
Pain Management pocketcards	Börm Bruckmeier Publishing LLC	$4,99	Medical	US, UK, CA	iPad, iPhone	Pain
Pain Pal: chronic pain control	ZenZone Interactive Limited	$ 1,99	Medical	US	iPad, iPhone	Pain
Pain Scale - your digital log for chronic pain	odeesoft	$ 5,99	Medical	US	iPad, iPhone	Pain
Pain Scored	PATIENT PREMIER, LLC	Free	Medical	US, CA	iPad, iPhone	Pain
Pain Squad	The Hospital for Sick Children	Free	Medical	US, UK, CA	iPhone	Pain
Pain Toolkit	Advanced Digital Institute - Health	Free	Medical	US, UK, CA	iPad, iPhone	Pain
Pain Tracker	Terry Billingsley	Free	Health & Fitness	US	iPhone, iPad	Pain
Pain Tracker & Diary	Nanolume, LLC	$2,99	Medical	US	iPad, iPhone	Pain

Pain-Scale	BGU Medical Informatics Research Center	Free	Health & Fitness	US	iPhone	Pain
PainPoint – Prevent Work Injury	www.ohcow.on.ca	Free	Health & Fitness	US	iPad, iPhone	Pain
PainScale - Pain Tracker Diary	Boston Scientific	Free	Medical	US	iPhone	Pain
PainTrakr	Black Slate Software Inc.	Free	Medical	US, UK, CA	iPad, iPhone	Pain
RheumaBuddy	Daman P/S	Free	Medical	UK	iPhone	Rheumatoid arthritis
Signs & Symptoms Low Back Pain	BuiltByDoctors	Free	Medical	US	iPad, iPhone	Low back pain
Symdir - symptom diary made easy	SILECI	Free	Medical	CA	iPad, iPhone	Pain
Symple Symptom Tracker	Symple Health, Inc.	Free	Medical	US, UK, UK	iPhone	Pain
Symptom Diary	Stuart Harris	Free	Medical	UK	iPhone	Pain
Symptom Tracker by TracknShare	Track & Share Apps, LLC	$ 6,99 CA	Medical	CA	iPad, iPhone	Pain
Symptom Tracker: Pain Headache	Telemedhome,LLC	Free	Health & Fitness	US, CA	iPhone, iPad	Headache
Symptom Tracker: Pain History	SmoothMobile, LLC	Free	Health & Fitness	US	iPhone	Pain
Track My Pain	Paul Brungardt	Free	Health & Fitness	US	iPhone	Pain
TRACK + REACT	Arthritis Foundation	Free	Health & Fitness	UK, CA	iPhone	Pain
TracknShare LITE	Track & Share Apps, LLC	Free	Medical	US, UK, CA	iPad, iPhone	Pain
Visual Scale (painometer, pain level measuring)	Bit Genoma Digital Solutions SL	Free	Medical	US, UK, CA	iPad, iPhone	Pain

Table S8. Pain apps available in AppStore for iOS (Vionza)

App Name	Developer	Pain problem (condition)
Back Pain Tracker	PEERClinic for Back Pain and Spine Surgery	Back pain
Essential Pain Management	Qworks Technologies Pvt Ltd	Pain
FibroMapp Pain Manager +	Bodymap Apps	Fibromyalgia
Manage My Pain	ManagingLife	Pain
Manage My Pain Pro	ManagingLife	Pain
Migraine Buddy - The Migraine and Headache tracker	Healint	Migraine and Headache
My Pain Diary	DamoLab LLC	Pain
Ouchie: your pain management companion	Ouchie	Pain
Pain Assessment Tool for children	Bcom	Pain
Pain Companion	Sanovation AG	Pain
Pain Diary - Pain Management Log	Stay fit with Samantha	Pain
Pain Diary (Privacy Friendly)	SECUSO Research Group	Pain
Pain Diary & Forum CatchMyPain	Sanovation AG	Pain
Pain Log	Raul R.	Pain
Pain Log - Pain Tracker	Skillo Apps	Pain
Pain Scale VAS		Pain
Pain Scored	Patient Premier	Pain
Pain Tracker & Diary	Nanonlume LLC	Pain
Pain Tracker HD	AppYourWay	Pain
PainScale - Free Chronic Pain Tracker Diary	Boston Scientific Inc	Pain
Track My Pain	King Louie Apps	Pain
WebMD: Check Symptoms, Find Doctors, & Rx Savings	WebMD LLC	Various health conditions

Table S9. Pain apps available in Google Play for Android

App Name	Developer	Country	Language	Platforms	Price	Condition
CatchMyPain-Pain Diary	Sanovation AG; Software Evolution and Architecture Lab (SEAL) Gruppe, Instituts für Informatik, Universität Zürich (http://www.sanovation.com)	Switzerland	English, French, German, Spanish	Android, iOS, web	Free. Various in app purchases	Pain
FMAUKFibroMapp	BodyMap Apps, UK http://www.bodymapapps.com	United Kingdom	English	Android, iOS	£2,99	Fibromyalgia.
Headache Diary (ecoHeadache)	ecoTouchMedia (Jae-Ung Yi)	Germany	English / French / German / Italian / Japanese / Korean / Spanish	iOS, web	£1.91	Headache
Headache Diary Lite	Froggyware GmbH http://www.froggyware.com	Germany	English / French / German / Italian / Spanish	Android, web	Lite version free, Android full version: £1.99	Headaches or migraines.
iheadache	BetterQOL.com, USA http://www.betterqol.com	United States	English	iOS, web	Lite' version free on Apple; Apple full version: £3.20	Headache
Manage My Pain Lite	ManagingLife http://www.managinglife.com	Canada	English	Android, web	Free, Pro version £2.99	Pain.

My Pain Diary: Chronic Pain Management	Damon Lynn, USA<br>http://www.damonlynn.com	United States	English	iOS	£3.99	Pain
myIBD	Karen Frost; Dr. Johan Van Limbergen; Meaghan Wright (and Ritchie Hwang), Canada<br>http://www.sickkids.ca/AboutSickKids/Newsroom/Past-News/2011/SickKids-mobile-app-helps-IBD-patients.html	Canada	English	iOS, web	Free	Inflammatory Bowel Disease (IBD).
Pain Care	Ringful Health, USA<br>http://www.ringfulhealth.com	United States	English	Android, iOS	Free	Chronic pain or sports injuries.
RheumaTrack	Nicole Derouaux of Mutterelbe Medical UG, Germany<br>http://www.mutterelbe.de	Germany	English / French / German / Spanish / Turkish	Android, iOS	Free	Rheumatism
SmallTalk Intensive Care	Lingraphicare America Inc, USA<br>http://www.aphasia.com	United States	English	iOS, web	Free	Pain
SmallTalk Pain Scale	Lingraphicare America Inc, USA<br>http://www.aphasia.com	United States	English	iOS	Free	Pain
WebMD	WebMD LLC, USA<br>http://www.webmd.com/mobile	United States	English	Android, iOS	Free	Pain

Table S10. Pain apps reported in myhealthapps.net

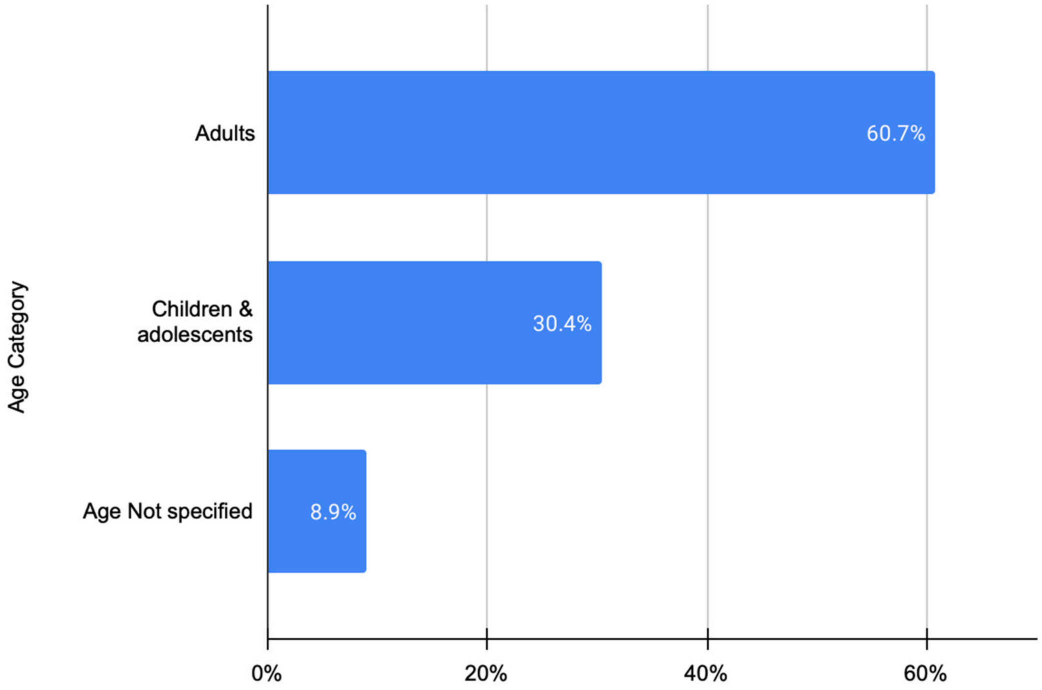

Figure S1. Target population (age) for selected articles

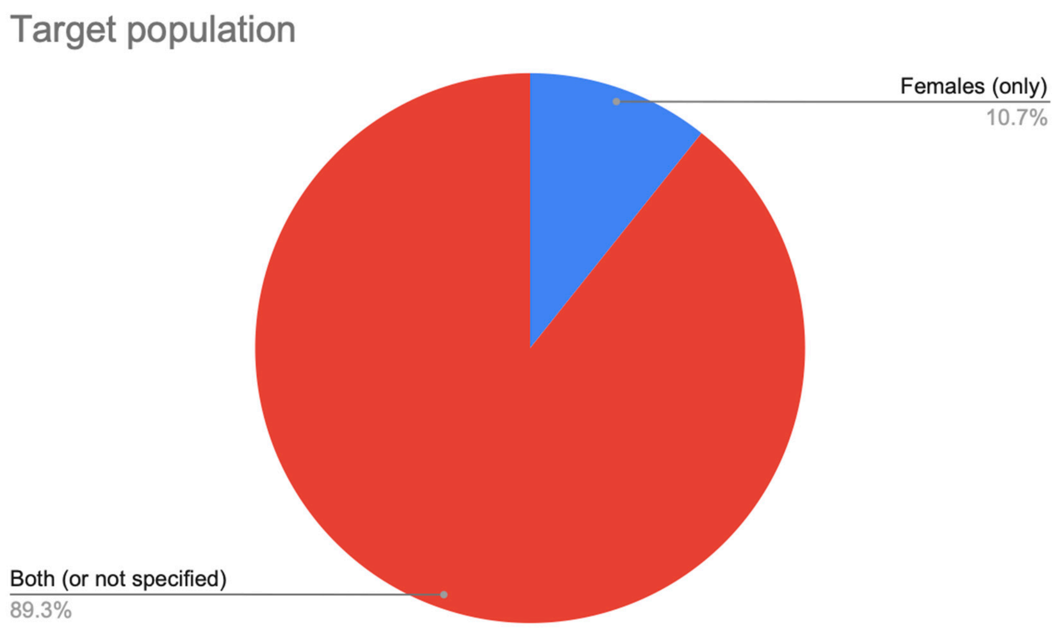

Figure S2. Target population (gender) for selected articles

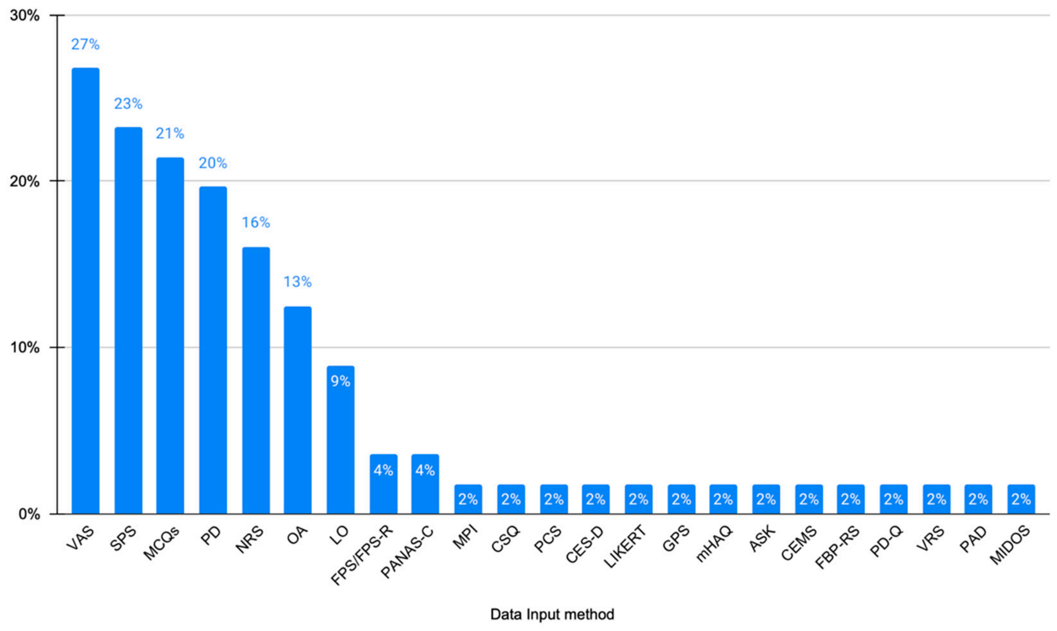

Figure S3. Data input method for pain assessment (articles)

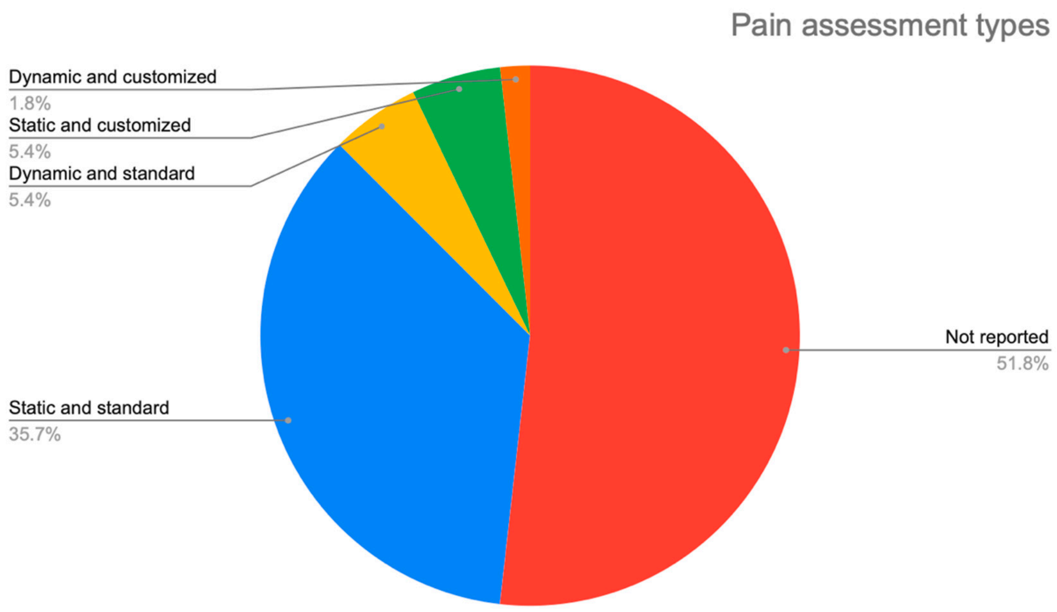

Figure S4. Types of pain assessment (articles)

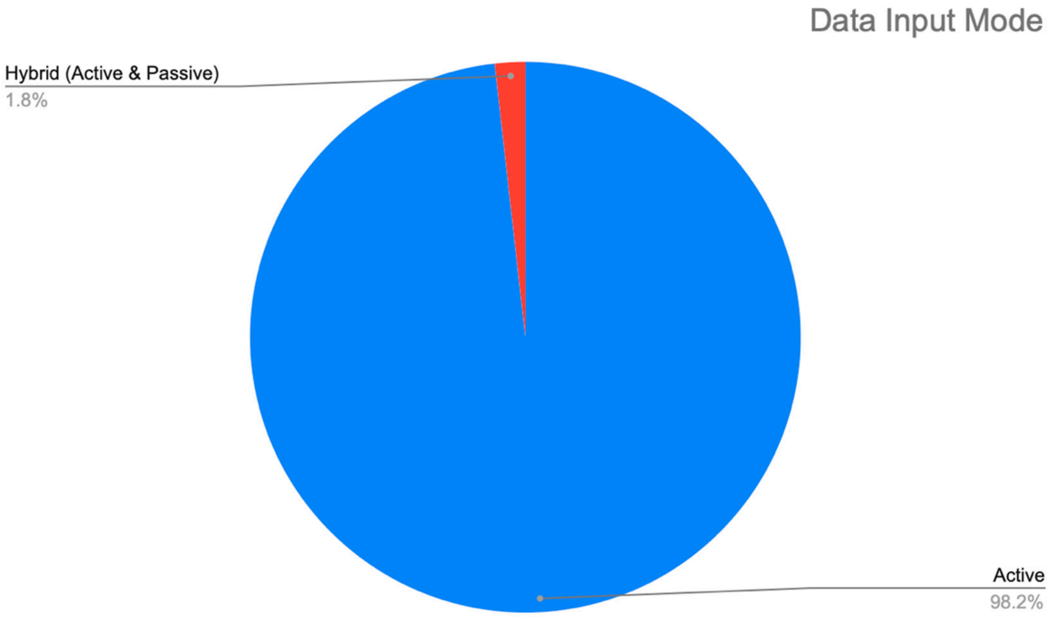

Figure S5. Types of data input mode (articles)

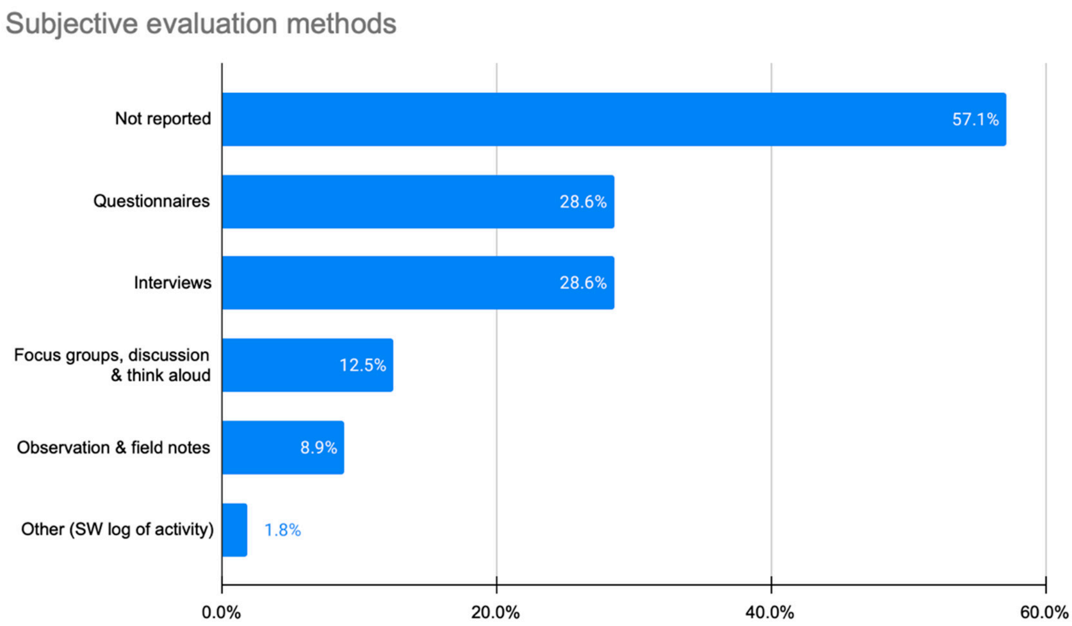

Figure S6. Subjective evaluation methods (articles)

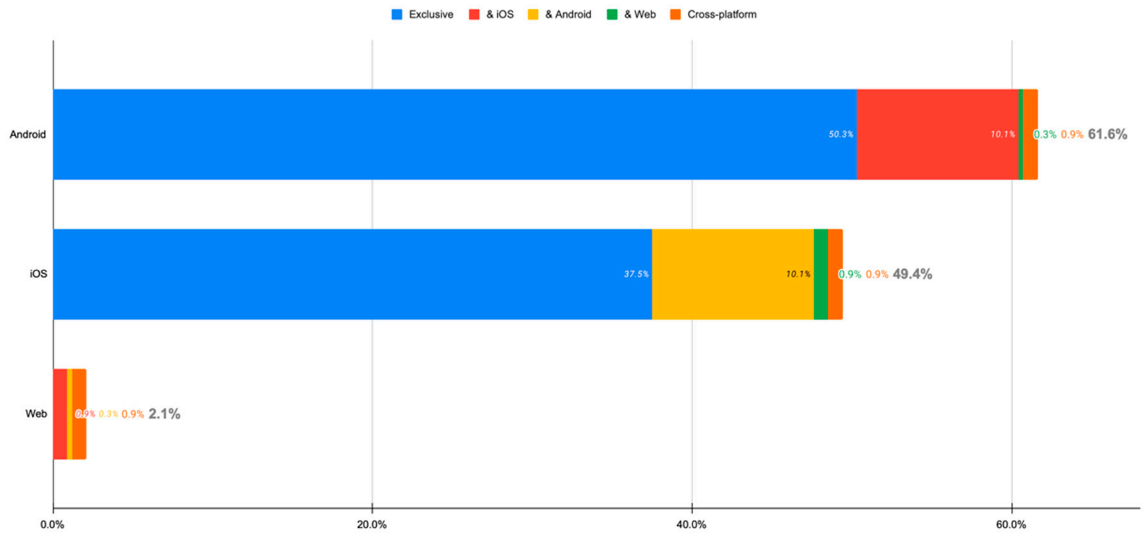

Figure S7. Target platform for commercial pain management apps

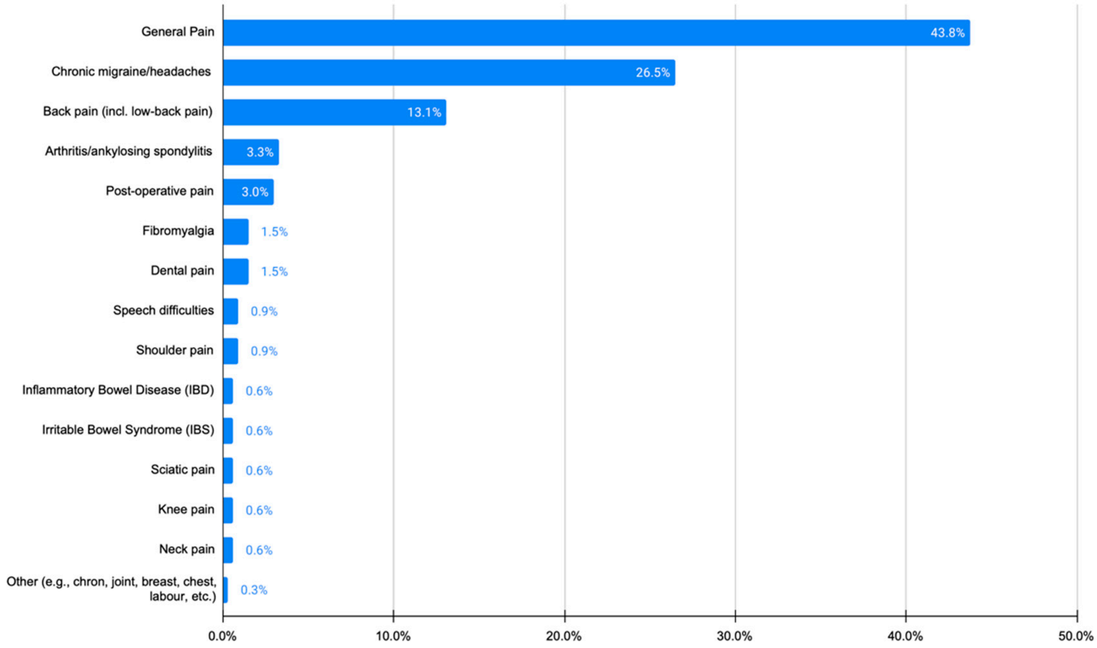

Figure S8. Target condition or pain type for commercial pain management apps

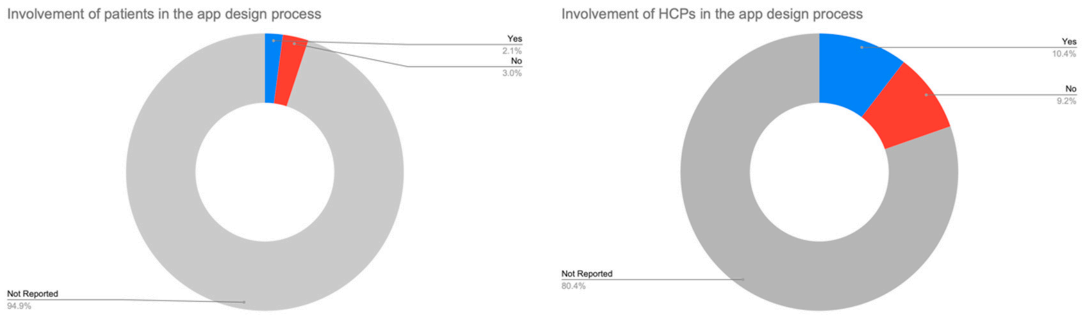

Figure S9. Design methodology for commercial pain management apps
